# Antioxidant Activity and Mechanism of Resveratrol and Polydatin Isolated from Mulberry (*Morus alba* L.)

**DOI:** 10.3390/molecules26247574

**Published:** 2021-12-14

**Authors:** Ziwei Li, Xiaoman Chen, Guo Liu, Jun Li, Jinglin Zhang, Yong Cao, Jianyin Miao

**Affiliations:** 1 Guangdong Provincial Key Laboratory of Nutraceuticals and Functional Foods, College of Food Science, South China Agricultural University, Guangzhou 510642, China; liziwei@stu.scau.edu.cn (Z.L.); e0554125@u.nus.edu (X.C.); liuguo@scau.edu.cn (G.L.); pretty_lj@163.com (J.L.); caoyong2181@scau.edu.cn (Y.C.); 2 Key Laboratory of Brewing Molecular Engineering of China Light Industry, Beijing 100048, China; 3 Guangxi Key Laboratory of Chemistry and Engineering of Forest Products, Guangxi University for Nationalities, Nanning 530006, China

**Keywords:** mulberry, resveratrol, polydatin, extraction, antioxidant activity, mechanism

## Abstract

Natural stilbenes have unique physiological effects, such as anti-senile dementia, anti-cancer, anti-bacterial, lowering blood lipid, and other important biological functions, which have attracted great attention from scholars in recent years. In this study, two stilbene compounds, resveratrol (RES) and polydatin (PD), were isolated from Mulberry (*Morus alba* L.), and their antioxidant activity and mechanism were investigated. The results showed that the contents of RES and PD in mulberry roots were 32.45 and 3.15 μg/g, respectively, significantly higher than those in mulberry fruits (0.48 and 0.0020 μg/g) and mulberry branches (5.70 and 0.33 μg/g). Both RES and PD showed high antioxidant potential by DPPH, ABTS free-scavenging methods, and ORAC assay, and provided protection against oxidative damage in HepG2 cells by increased catalase (CAT) activity, superoxide dismutase (SOD) activity, and Glutathione (GSH) content, and decreasing generation of reactive oxygen species (ROS), lactate dehydrogenase (LDH) level, and malondialdehyde (MDA) content. Therefore, RES and PD treatment could be effective for attenuating AAPH-induced oxidative stress in HepG2 cells. This study will promote the development and application of stilbene compounds. Furthermore, the RES and PD could be used as antioxidant supplements in functional foods, cosmetics, or pharmaceuticals, contributing to health improvement.

## 1. Introduction

There is an increasing demand for the beneficial effects of natural-source functional products and their biologically active molecules [1]. Functional ingredients such as polyphenols, bioactive peptides, probiotics, vitamins, and minerals are directly added to various foods to enhance their potential to prevent or control various diseases [2,3]. Among these bioactive compounds, plant-based functional foods have received significant attention in the food industry in recent times. More than 10,000 bioactive phytochemicals have been identified from plant-derived foods [4]. However, looking for some highly active phytochemicals from a wide range of sources is still the focus of research in the field of functional foods [5,6].

Resveratrol (trans-3,5,4′- trihydroxy trans stilbene, RES) is a polyphenol compound found naturally in plant-based foods, which is considered as a potential functional-feed additive [7]. The basic structure of RES is composed of two phenol rings linked to each other by an ethylene bridge, which can be divided into two isomeric forms of *trans* resveratrol and *cis* resveratrol. It is usually found in plants in *trans* resveratrol form, and when taken orally, trans-resveratrol can be rapidly transformed to the biologically more active form of dihydro-resveratrol [8]. As RES has many potential health benefits for human health, such as anti-cancer, anti-inflammatory, anti-diabetic activities, hepato-protection, and inhibition of platelet aggregation [9,10], its application as a biologically active ingredient has received extensive attention. In fact, RES is becoming a popular ingredient in dietary supplements and vitamin formulas, and it is expected to double in the global market in the next decade [11]. Furthermore, Polydatin (3,4′,5-trihydroxystibene-3-β-mono-D-glucoside, PD), as the most abundant derivative of RES in nature, has attracted attention in the pharmaceutical and functional food industries ascribed to its higher oral bioavailability [12]. Moreover, in nature, PD tends to be more abundant than RES [13]. Therefore, there are many studies devoted to converting PD into RES, but ignore the functional properties of PD compounds [14]. It is worth noting that PD also performs well in many biological functions, such as anti-atherosclerosis [15], improving heart function [16], reducing cholesterol [17], and protecting against liver injury [18]. Moreover, PD has high water solubility and metabolic stability [19]. Among the numerous pharmacological effects, antioxidant effect is undoubtedly the most important. As antioxidant activity is related to a variety of physiological and metabolic activities, it is the most important prevention mechanism of the human body [20]. However, the extraction of natural RES and PD from plant resources and the comparative analysis of their activities have rarely been reported.

In the present study, RES and PD were isolated and identified from Mulberry (mulberry fruits, mulberry branches, and mulberry roots). The antioxidant activities of RES and PD were comprehensively evaluated by in vitro chemical and cell models. Finally, through establishing a cellular oxidative stress model induced by AAPH, the antioxidant protection mechanisms of RES and PD were studied.

## 2. Results

### 2.1. Separation and Identification of RES and PD

**Figure 1:**
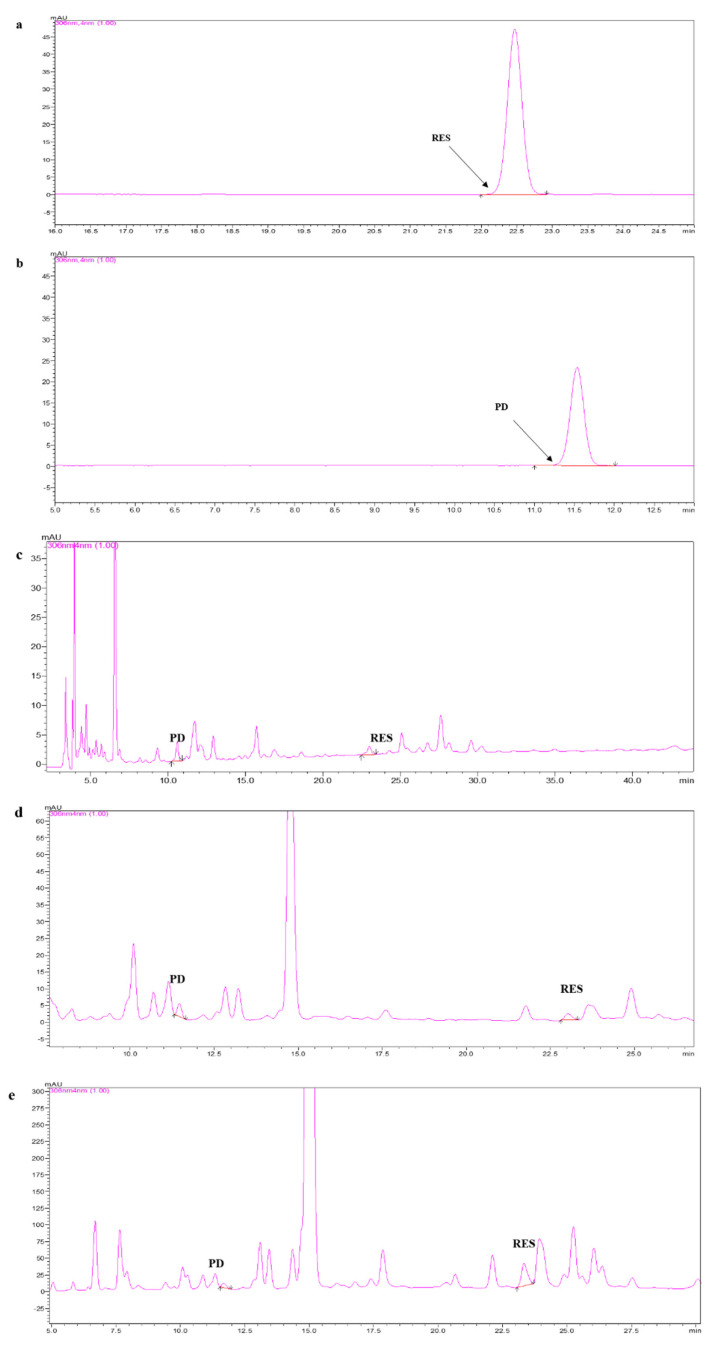
Isolated and purified RES and PD from mulberry fruits, mulberry branches, and mulberry roots. (**a**) The HPLC chromatogram of the RES standard compound. (**b**) The HPLC chromatogram of the PD standard compound. (**c**) The HPLC chromatogram of the RES and PD from mulberry fruits. (**d**) The HPLC chromatogram of the RES and PD from mulberry branches. (**e**) The HPLC chromatogram of the RES and PD from mulberry roots.

The extracts of RES and PD were firstly extracted from mulberry fruits, mulberry branches, and mulberry roots, respectively. Then, taking RES and PD standards as analytical references, the extracts were further separated and analyzed by high-performance liquid chromatography (HPLC). The chromatogram of the RES and PD standards were firstly observed and their retention time was 22.466 min (Figure 1a) and 11.529 min (Figure 1b), respectively. By comparing with the retention time of the RES and PD standards, the RES and PD were further purified from the extracts of mulberry fruits (Figure 1c), mulberry branches (Figure 1d), and mulberry roots (Figure 1e). The contents of RES were calculated, and we were surprised to find that RES in mulberry roots (32.45 μg/g) was the highest, which was 5.7 times and 68.2 times that in mulberry branches (5.70 μg/g) and mulberry fruits (0.48 μg/g) (Table 1). Similarly, the content distribution of PD in Mulberry was also measured. We found that the content of PD in mulberry roots (3.15 μg/g) was the highest, which was 9.6 times and 1576.7 times that in mulberry branches (0.33 μg/g and mulberry fruits (0.0020 μg/g) (Table 1). Interestingly, RES and PD were most abundant in mulberry roots, and the content of RES was significantly higher than that of PD, which was 10.3 times that of PD.

**Figure 2:**
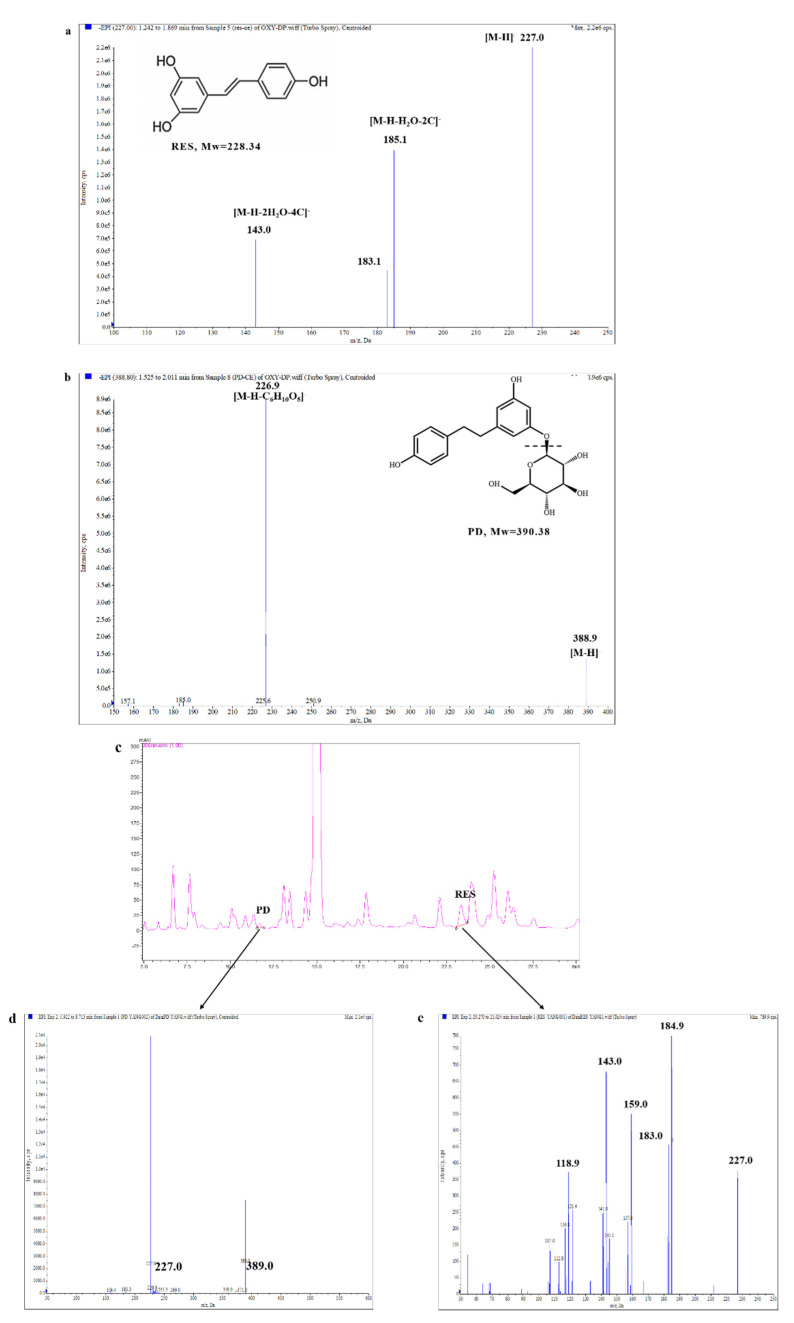
Identification of RES and PD from mulberry roots by UHPLC-ESI-MS/MS. (**a**) The fingerprint mass spectrum of RES standard. (**b**) The fingerprint mass spectrum of PD standard. (**c**) The HPLC chromatogram of the RES and PD from mulberry roots. (**d**) The MS2 spectrum of PD isolated from mulberry roots. (**e**) The MS2 spectrum of RES isolated from mulberry roots.

Based on the above analysis, RES and PD from mulberry roots were further identified by UHPLC-ESI-MS/MS (Figure 2). The fingerprint mass spectra of RES and PD standards were obtained for the first time (Figure 2a,b), which can be used as a reference for the identification of RES and PD from mulberry roots. It can be seen from Figure 2d that the precursor ion peak [M − H]^−^ of PD was detected at *m*/*z* 389.0, and its product ion fragment at *m*/*z* 227.0. Furthermore, the precursor ion peak [M − H]^−^ of RES was detected at *m*/*z* 227.0, and its product ion fragment at *m*/*z* 143.0 and *m*/*z* 184.9. (Figure 2e). Convincingly, these spectral data were consistent with those of the standards, and are also consistent with the data described in the literature [21,22]. This means that RES and PD were successfully isolated and identified from mulberry root.

### 2.2. In Vitro Antioxidant Activities

#### 2.2.1. DPPH Free Radical-Scavenging Activity

**Figure 3:**
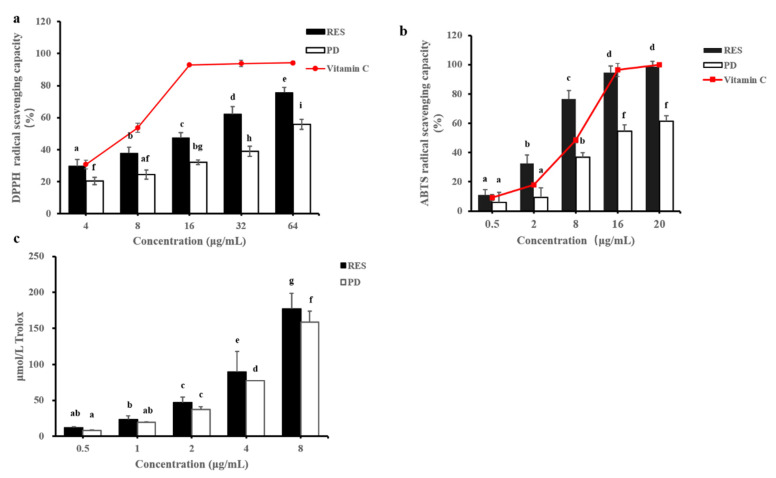
The in vitro antioxidant activities of RES and PD. (**a**) DPPH radical-scavenging activity. (**b**) ABTS radical-scavenging activity. (**c**) ORAC values. Data are shown as mean ± SD (*n* = 3). Different superscript characters indicate significant difference at *p* < 0.05 level within the same row.

The DPPH free radical-scavenging activities of the RES and PD are shown in Figure 3a. Both samples showed DPPH radical-scavenging activity in a dose-dependent manner. The RES showed higher inhibition ability of DPPH free radical oxidation than PD, suggesting that RES had higher antioxidant activity. The scavenging rate of RES on DPPH free radicals increased from 29.56% to 75.63%, an increase of 1.51 times. Furthermore, the scavenging rate of DPPH free radicals by PD increased from 20.54% to 55.80%, an increase of 1.72 times. This shows that although the antioxidant activity of PD was not as strong as that of RES, when the same concentration was increased, the antioxidant activity of PD has a better enhancement effect. Based on the IC_50_ values, the antioxidant activities of RES (IC_50_ = 15.54 µg/ mL) and PD (IC_50_ = 54.35 µg/ mL) were weaker than Vitamin C (IC_50_ = 6.35 µg/ mL), but they were stronger than those of traditional medicine *Pometia pinnata* (IC_50_ = 92 µg/mL) [23] and *Lygodium circinnatum* (IC_50_ = 143.76 µg/mL) [24].

#### 2.2.2. ABTS Free Radical-Scavenging Activity

This method was determined by evaluating the decolorization ability of some phenols and flavonoids from plants after reacting with ABTS free radicals [25]. The ABTS free radical-scavenging activities of RES, PD, and Vitamin C are shown in Figure 3b. According to the results, all compounds examined showed dose-dependent ABTS^·+^ radical-scavenging activity, but RES exhibited higher ABTS^·+^ radical-scavenging activities than the PD and Vitamin C. In addition, RES, PD, and Vitamin C exhibited IC_50_ values of 2.86, 13.44, and 5.18 μg/mL, respectively. Compared with the results of DPPH, RES has a very different scavenging efficiency on ABTS free radicals, which may be caused by different mechanisms. In the DPPH experiment, the hydrogen supply capacity of a compound determines the scavenging effect of free radicals, while the scavenging effect of ABTS^·+^ is determined by the scavenging effect of proton free radicals by giving electrons [26]. Therefore, we speculate that RES plays an antioxidant role by removing a proton from the hydroxyl group and giving it to free radicals to form stable phenoxy groups. This view was supported by the research of Das, A. K et al. [27]. In addition, as a potent antioxidant, *Camellia sinensis* tea was also more sensitive to ABTS^·+^ scavenging ability than DPPH free radical-scavenging ability [28].

#### 2.2.3. ORAC (Oxygen Radical Absorption Capacity)

The ORAC experiment is to determine the hydrophilic chain-breaking antioxidant capacity of the compound [29]. The method was highly related to biology because it uses physiological oxidant as reactant and reacts under physiological pH conditions [30,31]. The results for the assay are presented in Figure 3c. ORAC exhibited the same results as represented by DPPH and ABTS free radicals, showing a concentration-dependent manner, and the antioxidant activity of RES is always better than that of PD. When the RES concentration increased from 0.5 μg/mL to 8 μg/mL, the corresponding Trolox concentration increased from 11.97 μmol/L to 177.19 μmol/L, an increase of 14.80 times. The concentration of PD increased from 0.5 μg/mL to 8 μg/mL, and corresponding Trolox concentration increased from 7.87 μmol/L to 158.98 μmol/L, an increase of 20.20 times. This interesting phenomenon was consistent with the above DPPH assay results, indicating that the antioxidant activity of PD was more affected by concentration than RES. Furthermore, the ORAC value of each compound was expressed by Trolox equivalent (TE), and the ORAC value of RES was 23.12 μmol TE/g, and that of PD was 18.67 μmol TE/g. These data were similar to honey (18.48 μmol TE/g) [32] and significantly higher than the ethanolic leaf extract of *A. trifoliatusthe* (9057.29 μmol TE/100 g) [33]. The result indicated that RES and PD exhibited very good antioxidant activity.

### 2.3. CAA

**Figure 4:**
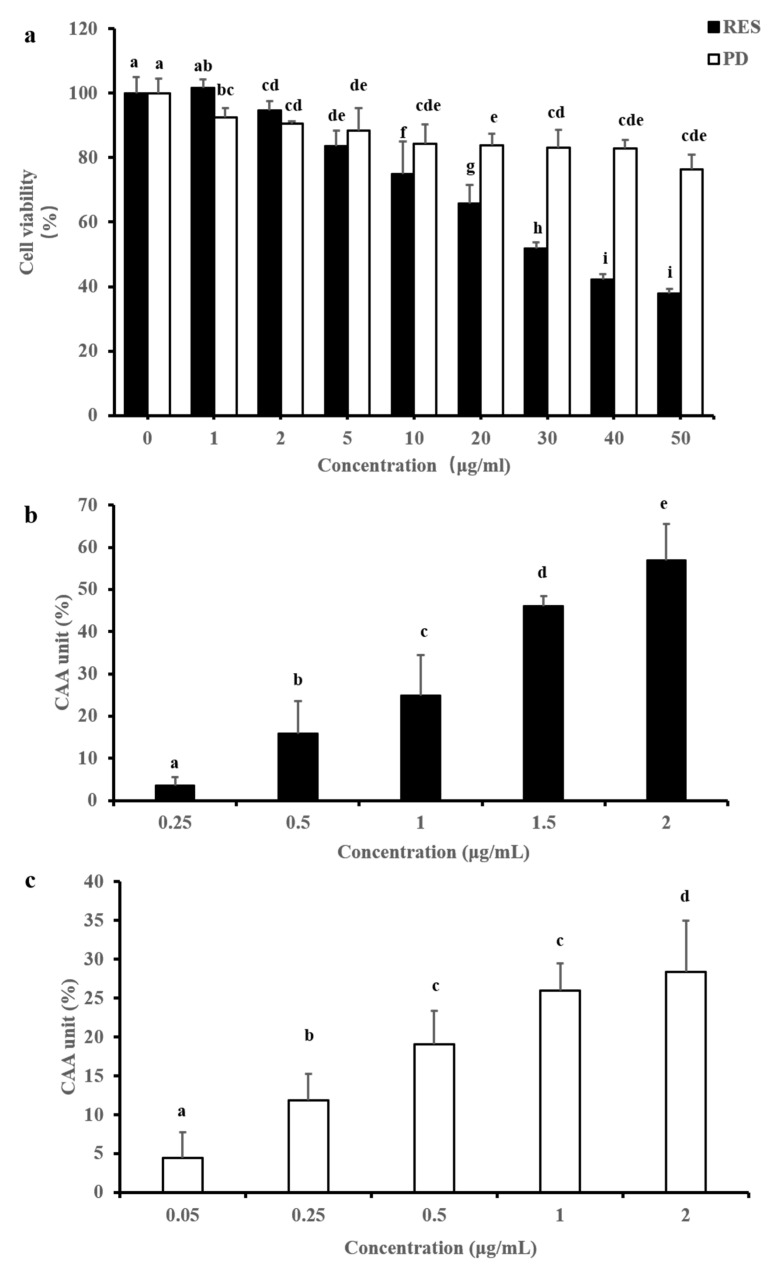
Determination of the cellular antioxidant activities of RES and PD. (**a**) The cytotoxic effects of RES and PD on HepG2 cells. (**b**) Cell antioxidant activity of RES at different concentrations. (**c**) Cell antioxidant activity of PD at different concentrations. Data are shown as mean ± SD (*n* = 3). Different letters on top of the bars denote significant difference (*p* < 0.05) among different concentrations.

The cytotoxicity and antioxidant activity effects of RES and PD on HepG2 cells are shown in Figure 4. As shown in Figure 4a, 0–50 μg/mL of RES and PD have different degrees of toxic effects on cells. In general, the cytotoxicity of PD to cells was slight, and the cell viabilities of the two compounds below 2 μg/mL were more than 90%, indicating that the concentration range does not affect the viability of cells. Therefore, 0–2 μg/mL RES and PD were selected to further evaluate the intracellular antioxidant activity. The two compounds at different concentrations showed the ability to quench peroxyl radical-induced oxidation; furthermore, both exhibit a dose-dependent activity. In Figure 4b, the CAA unit of the RES was significantly increased from 3.60% to 56.84%, and its EC_50_ and CAA values were calculated as 1.66 μg/mL and 331.80 μmol QE/100 g compound, respectively. Similarly, the CAA unit of PD increased significantly from 4.48% to 28.43% (Figure 4c). In addition, the CAA unit of PD did not reach 50% in the measured dose, so its EC_50_ value could not be calculated and CAA values were also not available. Therefore, consistent with the in vitro chemical antioxidant results, the antioxidant activity of RES in cells was also stronger than that of PD. Moreover, the cellular antioxidant effect of RES was significantly stronger than that of blueberry polyphenols [34] and cabbage [35], which were rich in anthocyanins.

### 2.4. Protective Effects of RES and PD Compounds on HepG2 against AAPH-Induced Oxidative Stress

**Figure 5:**
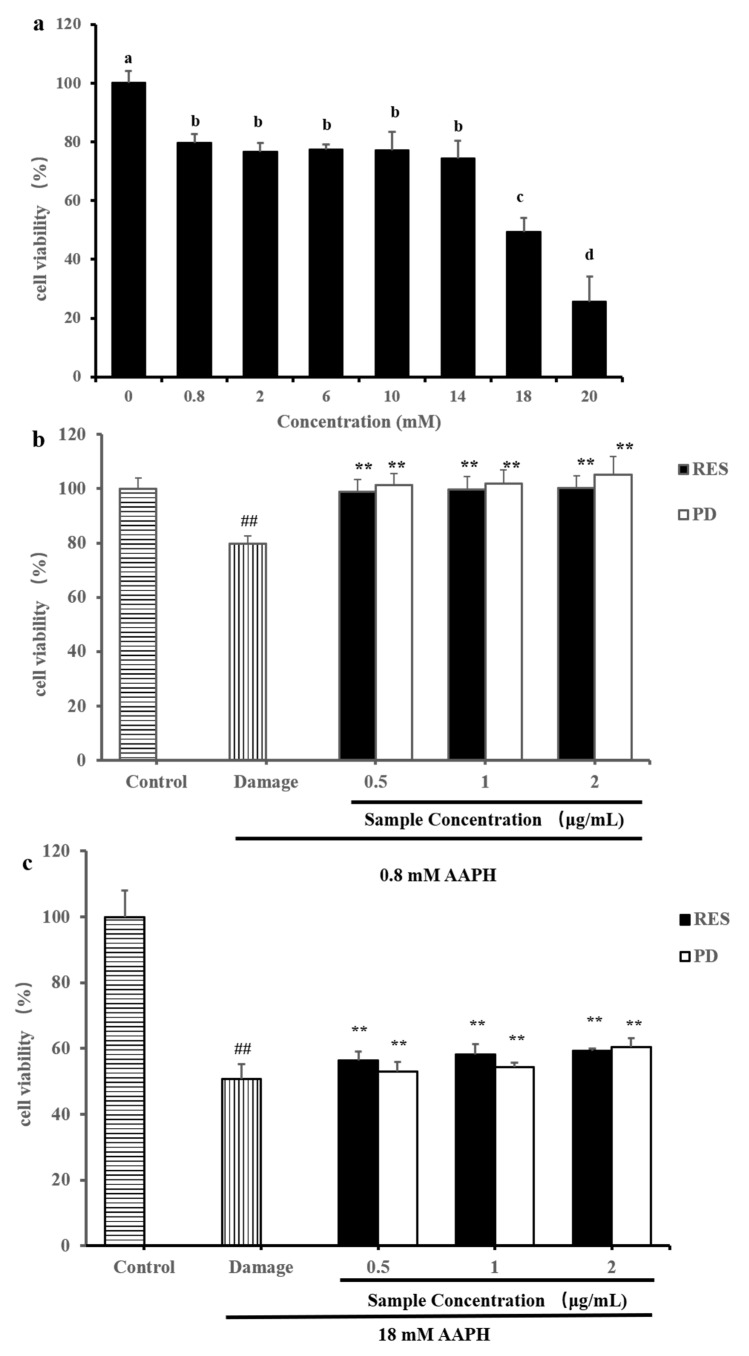
The protective effects of RES and PD against AAPH-induced oxidative stress. (**a**) Cell viability of AAPH-induced HepG2 cells. (**b**) The protective effects of RES and PD against 0.8 mM AAPH-induced oxidative stress in HepG2 cells. (**c**) The protective effects of RES and PD against 18 mM AAPH-induced oxidative stress in HepG2 cells. Data are shown as mean ± SD (n = 3). Different letters on top of the bars denote significant difference (*p* < 0.05) among different concentrations. ^##^ *p* < 0.01 when compared to the control group; ** *p* < 0.01 when compared to the damage group.

AAPH could generate peroxyl radicals and be used as an initiator to induce the oxidative stress reaction of cells, which was a classic oxidative damage model [36]. In Figure 5a, the cells were treated with different concentrations of AAPH to construct a cell oxidative damage model. After exposure to 0.8–20 mM AAPH for 24 h, the cell viability rates were 79.56%, 76.68%, 77.46%, 77.18%, 74.46%, 49.37%, and 25.70%, respectively. According to the cell viability, we selected two different degrees of cell-damage effects for further experiments, which were treated with 0.8 mM AAPH (cell viability rate was 79.56%) and 18 mM AAPH (cell viability rate was 49.37%).

As shown in Figure 5b, after exposure to 0.8 mM AAPH for 24 h, cell viability was significantly decreased compared to the control group. However, pretreatment with RES or PD attenuated AAPH-induced cell death. Compared with the damage group, the RES of 0.5–2 μg/mL significantly increased the cell viability rate from 79.65% to 99.02%, 99.60%, and 100.25%, respectively. Similarly, compared with the damage group, the PD of 0.5–2 μg/mL significantly increased the cell viability rate from 79.65% to 101.33%, 101.90%, and 105.10%, respectively. These results indicate that pretreatment with 0.5–2 μg/mL RES or PD significantly alleviated 0.8 mM AAPH-induced oxidative damage in HepG2 cells, and even restored to normal level. Moreover, the protective effects of RES and PD on the viability of 18 mM AAPH-treated HepG2 cells were further evaluated. As shown in Figure 5c, the HepG2 cells treated with 18 mM AAPH for 24 h revealed about 50.72% cell viability rate. The cell survival rate was only 1.35% lower than that of the oxidative damage model, indicating that the model was stable. As shown in Figure 5c, pretreatment of the cells with RES or PD for 24 h followed by AAPH markedly increased the cells viability in a dose-dependent manner. In particular, 0.5, 1, and 2 μg/mL RES restored cell viability up to 56.39%, 58.25%, and 59.27%, respectively. Furthermore, 0.5, 1, and 2 μg/mL PD restored cell viability up to 52.89%, 54.43%, and 60.40%, respectively. Interestingly, based on the above results, we found that RES and PD showed better protective effects when the cells were treated to a lower degree of oxidative damage (cells were treated with 0.8 mM AAPH). Surprisingly, in this degree of damage cells (0.8 mM AAPH treatment), PD exhibited better protective effect than RES. Therefore, the protective mechanism of RES and PD against AAPH-induced oxidative stress needs further study, which may be related to the structural difference and the inhibition of reactive oxygen species (ROS) production [37,38].

### 2.5. Effects of RES and PD on ROS Level in AAPH-Induced HepG2 Cell Damage

**Figure 6:**
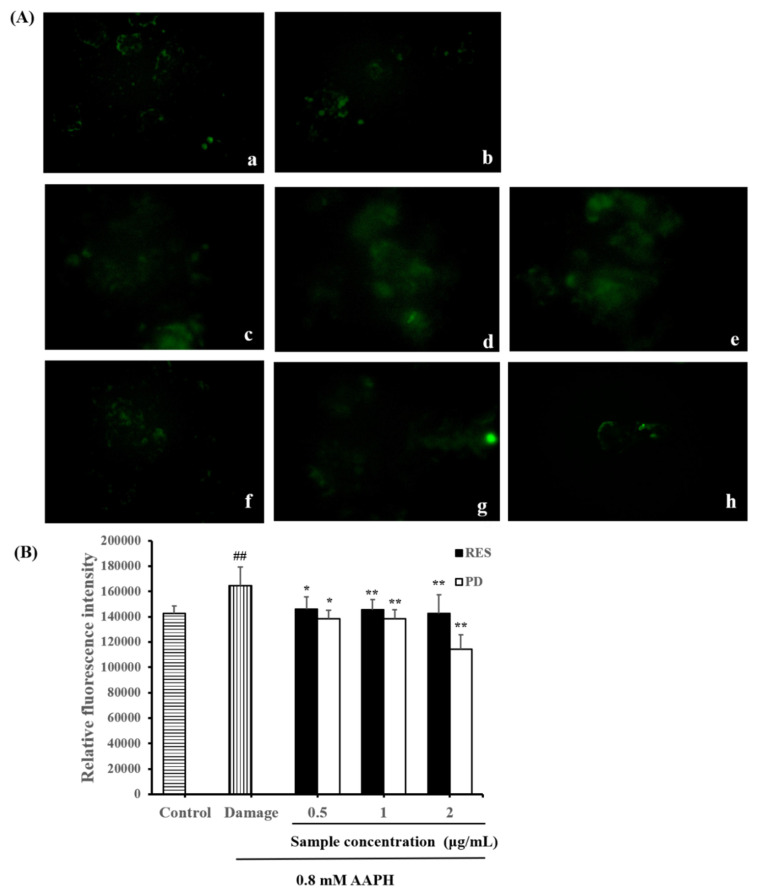
The intracellular ROS-scavenging abilities of RES and PD under conditions of AAPH-induced oxidative stress in HepG2 cells. (**A**): The fluorescence images of the cellular ROS-inhibitory effects of RES and PD in AAPH-induced HepG2 cells. (**a**) Control group without AAPH and pretreatment with samples. (**b**) Damage-group pretreatment with 0.8 mM AAPH. Images (**c**–**e**) show the effects of 0.5, 1, and 2 μg/mL RES pretreatment add 0.8 mM AAPH treatment, respectively. Images (**f**–**h**) show the effects of 0.5, 1, and 2 μg/mL PD pretreatment add 0.8 mM AAPH treatment, respectively. (**B**): The fluorescence intensity value of the cellular ROS-inhibitory effects of RES and PD in AAPH-induced HepG2 cells. Data are shown as mean ± SD (*n* = 3). ^##^ *p* < 0.01 when compared to the control group; * *p* < 0.05 when compared to the damage group, ** *p* < 0.01 when compared to the damage group.

The inhibitory effects of different RES and PD concentrations on ROS production in 0.8 mM AAPH-treated HepG2 cells was further determined (Figure 6). DCFH-Da was used to evaluate the ROS production during oxidative stress. DCFH-Da can be deacetylated in the cells and react with free radicals (mainly hydrogen peroxide) to form its fluorescent product, DCF [39,40]. The level of intracellular fluorescence was proportional to the degree of oxidation. In Figure 6A, the images (a) to (f) clearly reveal that AAPH in the cell induces the generation of ROS and produces fluorescence, while RES and PD reduce the production of ROS, and the fluorescence gradually weakens. As shown in Figure 6B, after 24 h of exposure to 0.8 mM AAPH, relative fluorescence intensity in HepG2 cells significantly increased compared with those in the control group (*p* < 0.05). As expected, RES and PD pretreatment significantly attenuated the increases in the relative fluorescence intensity (*p* < 0.05). Compared with damage group, fluorescence intensity in RES-pretreated cells (0.5, 1, and 2 μg/mL) decreased by 11.15%, 11.42%, and 13.19%, respectively. Furthermore, PD-pretreated cells (0.5, 1, and 2 μg/mL) reduced fluorescence intensity by 15.90%, 15.91%, and 30.52%, respectively. Hence, our results indicate that RES and PD pretreatment can protect cells from oxidative damage by inhibiting the production of ROS. It has been reported that regulation of ROS level is an important way for the bioactive dietary compounds of plants with antioxidant potential to exert antioxidant effect [41].

### 2.6. Levels of Antioxidant Enzymes (SOD, LDH, CAT, and GSH) and MDA

**Figure 7:**
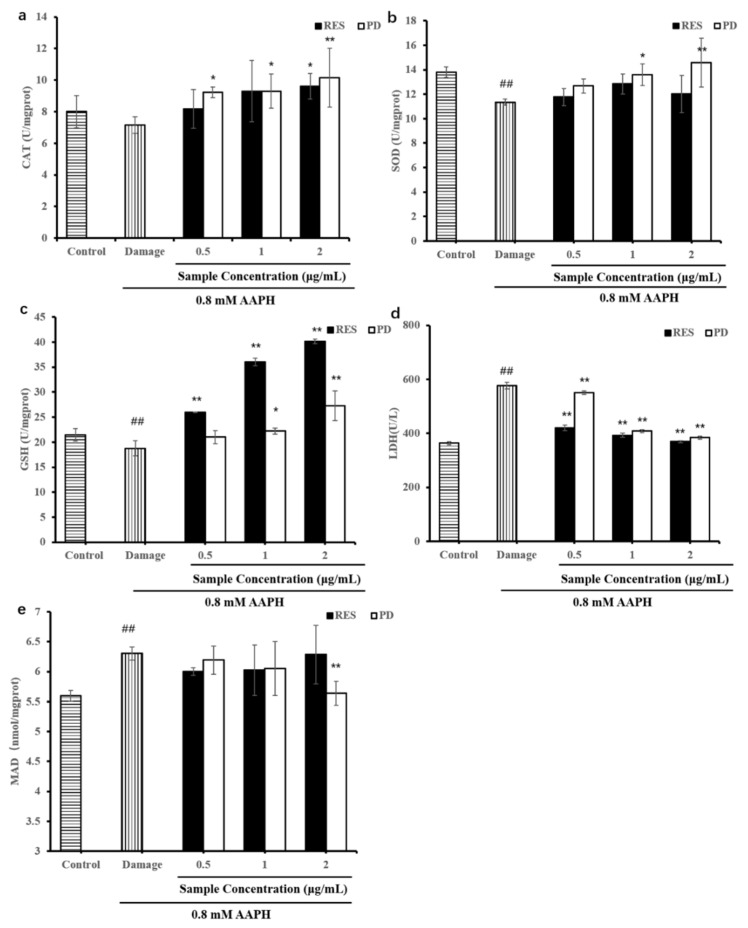
Effects of various concentrations of RES and PD on the AAPH-induced oxidative stress changes in the intracellular CAT (**a**), SOD (**b**), GSH (**c**), LDH (**d**), MDA (**e**) activities in HepG2 cells. Data are shown as mean ± SD (*n* = 3). ^##^ *p* < 0.01 when compared to the control group. * *p* < 0.05 when compared to the damage group, ** *p* < 0.01 when compared to the damage group.

Intracellular antioxidant enzyme systems, including CAT and SOD, play an important role in the defense against oxidative stress [42]. The change in antioxidant enzyme activity can be used as a reliable biomarker of antioxidant response. GSH is the most important endogenous small-molecule antioxidant that protects cells from chemically induced cytotoxicity [43]. To further understand the protective effects exerted by RES and PD, we examined changes in the activities of CAT, SOD, and GSH (Figure 7). Treatment of HepG2 cells with 0.8 mM AAPH for 24 h induced significant decreases in the activities of SOD and GSH content (*p* < 0.01). Treatment of HepG2 cells with 0.8 mM AAPH for 24 h induced significant decreases in the activities of SOD and GSH content (*p* < 0.01). Compared to the damage group, pretreatment of the cells with RES reversed the AAPH-induced decreases in CAT, SOD, and GSH levels, and 0.5–2 μg/mL RES increased CAT levels by 14.11%, 30%, and 34.36% (Figure 7a); increased SOD levels by 3.72%, 13.12%, and 5.96% (Figure 7b); increased GSH levels by 7.38%, 10.24%, and 11.39% (Figure 7c), respectively. Meanwhile, pretreatment with PD (0.5, 1, and 2 μg/mL) increased CAT, SOD activity and GSH levels in a dose-dependent manner. Compared with the damage group, the CAT activity in the PD-treated cells significantly increased by 28.74% to 41.86% (Figure 7a), the SOD activity and the GSH content increased by 11.67% to 28.48% (Figure 7b) and 5.59% to 6.89% (Figure 7c), respectively. Therefore, these increased levels of CAT, SOD, and GSH may be conferred to protection of RES or PD against AAPH-induced oxidative stress in HepG2 cells.

MDA and lactate LDH are indicators of cell damage and oxidative stress [44]. As shown in the Figure 7d, the LDH level of damage group was significantly higher than that of the control group (*p* < 0.01), which indicated that the cells were injured by AAPH. Compared with the damage group, all samples could effectively inhibit increases in LDH in AAPH-induced HepG2 cells (*p* < 0.01). Pretreatment with 2 μg/mL RES and PD showed the strongest inhibitory effect, which reduced LDH levels by 35.89% and 33.33%, respectively. In addition, compared with the damage group, all samples could also effectively inhibited increases in MDA in AAPH-induced HepG2 cells (Figure 7e). Pretreatment with 2 μg/mL PD showed the strongest suppressed the overproduction of MDA level decreasing to 5.64 nmol/mg protein. It is worth noting that PD was superior to RES in inhibiting MDA production, which once again indicates that PD can better protect cells from damage. Therefore, this can well explain the different protective effects of RES and PD compounds on HepG2 against AAPH-induced oxidative stress. The current results suggest that RES and PD were able to ameliorate oxidative stress and increase the expression of related antioxidant factors.

## 3. Materials and Methods

### 3.1. Chemicals and Materials

HPLC-grade acetonitrile and phosphoric acid were obtained from Sigma Chemical Co. (St. Louis, MO, USA). The resveratrol and polydatin compounds used as standards were purchased from Shanghai Yuanye Bio-Technology Co., Ltd. (Shanghai, China). 2,2-azinobis (3-ethylbenzothiazoline- 6-sulfonic acid) (ABTS) and 2,2-diphenyl-l-picrylhydrazyl (DPPH) were purchased from Sigma-Aldrich ((Milano, Italy).). Cell culture reagents, including high-glucose medium (DMEM), fetal bovine serum (FBS), penicillin-streptomycin, and Hanks’ balanced salt solution (HBSS), were purchased from Gibco Life Technologies (New York, NY, USA). 2′,7′-dichlorofluorescin diacetate (DCFH-DA) and,2,2′-Azobis(2-methylpropionamidine) (AAPH) were purchased from Sigma Chemical Co., Ltd. (St. Louis, MO, USA). Disposable 25 cm^2^ cell culture flasks and 96-well plates were obtained from Corning (Dalian, China). Organic membranes (0.45 μm) and other chemicals of analytical grade were purchased from Kemiou (Tianjin, China).

### 3.2. Extraction of RES and PD

The dried mulberry fruits, fresh mulberry branches, and mulberry roots were bought at Guangdong Baosang Yuan Health Food Co., Ltd. The plants were washed and dried in the oven (50 °C) until their weight was constant. Then, they were continuously pulverized in an Omni-mixer and ground to give 40-mesh-size powder.

The RES and PD were extracted from dried mulberry fruits, mulberry branches, and mulberry roots powders according to the previous method with a slight modification [45,46]. Based on the preliminary experiments, 3 g of powdered samples was added in 45 mL of 80% ethanol by the assistance of an ultrasonic bath for 60 min (two times, 30 min each time). The extracts were filtered with a Whatman filter paper and then the solvent was evaporated and concentrated to 10 mL by rotary evaporator. Afterwards, NaCl was added to the extract until it was saturated, and left to stand still at 4 °C for 10 h after filtration. Then, petroleum ether and ethyl acetate were added to the filtrate in order to remove impurities such as polysaccharides. After the extraction, the sample was dissolved in 50% methanol and the obtained extract was collected and stored in darkness at −20 °C for further analysis. The content of RES and PD was expressed in micrograms per gram of plant.

### 3.3. Separation and Analysis of RES and PD

The RES and PD were separated and quantified by HPLC (Shimadzu, Tokyo, Japan) and with an SPD-10A (V)vp ultraviolet detector. C18 column (250 mm × 4.6 mm × 5 μm, Ultimate, Shanghai, China) was used for the separation and the mobile phase was consisted of 0.1% phosphoric acid in water (A) and acetonitrile (B) and the flow rate was 1 mL/min. The mobile phase was programmed consecutively in a linear gradient as follows: 0–20 min (17% B–25% B); 20–40 min (25% B–28% B); 40–75 min (28% B–45% B); 75–85 min (45% B–95% B); 85–95 min (95% B–95% B); and 95–100 min (95% B–17% B). The multi-wavelength detector was monitored at 305 nm, the injection volume was 20 μL for each sample solution and the column temperature was maintained at 25 °C.

The identification of the RES and PD was carried out using an AB Sciex QTRAP^®^ 4500 UPLC-MS/MS (AB Sciex, Foster, CA, USA) triple quadrupole mass spectrometer equipped with an electrospray source operating in negative ionization mode. Multiple-reactions monitoring (MRM) was employed to monitor the transitions of the molecular ion ([M − H]^−^) of RES at *m*/*z* 227→185 and of PD at *m*/*z* 388.8→227. The scan range was *m*/*z* 100–500. Typical mass spectrometric operated conditions: source temperature, 550 ℃; ion spray voltage, −4500 V; ion source gas 1, 35 psi, ion source gas 2, 60 psi, curtain gas, 40, collision gas, medium, declustering potential (DP), −87.21 and −96.18 V; collision energy (CE), −25 V and −10 V.

### 3.4. In Vitro Antioxidant Activities

#### 3.4.1. DPPH Free Radical-Scavenging Activity

The DPPH free radical-scavenging activity was determined according to the method described by Miao et al. [47] with slight modifications. Briefly, 100 μL sample or standard (Vitamin C) with varying concentrations (4, 8, 16, 32, and 64 μg/mL) was added to the same volume of DPPH ethanolic solution (0.15 mM). The mixture was then shaken vigorously and incubated at room temperature for 30 min, and the absorbance was acquired at 517 nm. Then, radical-scavenging activity was calculated as follows:

(1) PPH radical scavenging activity (%)=[1−(As−Ab)/Ac]×100

Where: As is the absorbance in the presence of the sample; Ab is the absorbance in the presence of the blank; Ac is the absorbance of the control; IC_50_ was determined by probit regression using IBM’s Statistical Program for Social Sciences (SPSS, IBM, Armonk, NY, USA) analysis of variance (17.0).

#### 3.4.2. ABTS Free Radical-Scavenging Activity

The ABTS free radical-scavenging activity was determined by the method described by Miao et al. [47] with some modifications. First, ABTS^+^ stock solution was prepared by reacting 7 mM ABTS aqueous solution with 2.45 mM potassium persulphate at room temperature in the dark for 12–16 h. The solution was then diluted with distilled water to achieve the absorbance of 0.7 ± 0.02 at 734 nm. For the spectrophotometric measurement of the samples, 100 μL of diluted ABTS^+^ solution and 100 μL of sample were mixed. After incubation at room temperature for 10 min, the absorbance of the mixtures at 734 nm was measured. Vitamin C was used as a reference. ABTS free radical-scavenging activity was calculated using the same formula as that used to measure DPPH radical-scavenging activity. IC_50_ was determined by probit regression in SPSS (IBM, Armonk, NY, USA).

#### 3.4.3. ORAC Assays

The ORAC assay was performed as previously described by Miao et al. [47] with some modifications. Briefly, 50 μL RES or PD (0.5, 1, 2, 4, 8, 16 μg/mL) and 100 μL FL working solution (80 nM final concentration) were mixed in the black 96-well plate and preincubated for 2–3 min at 37 °C. Then, 50 μL APPH solution (153 mM final concentration) was then added, and the fluorescence was recorded for 60 min at excitation and emission wavelengths of 485 and 530 nm, respectively. Trolox, a water-soluble analogue of vitamin E, was used as a reference. The final results were calculated using the differences in areas under the FL decay curves between the blank and a sample and ORAC values were expressed as μmol Trolox equivalent (TE)/ g of RES or PD compound (μmol TE/g).

### 3.5. Cells Lines and Culture Conditions

The HepG2 cells (human hepatoma cell line) originated from the Cell Bank of Chinese Academy of Sciences (Shanghai, China). HepG2 cells were cultured in the high-glucose medium (DMEM) supplemented with 10% FBS, 1% MEM non-essential amino acid solution, 100 U/mL penicillin, and 100 mg/mL streptomycin in a humidified atmosphere with 5% CO_2_ and 95% room air at 37 °C.

#### 3.5.1. MTT Assay

The MTT assay was based on the protocol described by Miao et al. [47] with some modifications. Briefly, HepG2 cells were seeded in a 96-well plate of 1 × 10^4^ cells/well and incubated for 24 h. Then, the cells were treated with the samples at different concentrations (1, 2, 5, 10, 20, 30, 40, and 50 μg/mL). The cells were then incubated for an additional 24 h at 37 °C. Furthermore, 100 μL MTT (0.5 mg/mL final concentration) was added and incubated at 37 °C in the dark for 4 h. Finally, the MTT solution was replaced with dimethyl sulphoxide (DMSO) to solubilize the formazan crystals, and the absorption value was determined at 490 nm by using a microplate reader (EnSpire, Perkin Elmer, Singapore).

#### 3.5.2. CAA

Based on the method of Wolfe and Liu [39], HepG2 cells were seeded at 6 × 10^4^/well on a black 96-well plate in 100 μL of DMEM, excluding the outside wells. After incubation at 37 °C for 24 h, the DMEM was discarded and the cells were washed with PBS, and treated for 1 h in triplicate with 100 μL of RES or PD plus 25 µM DCFH-DA dissolved in DMEM. After the medium was discarded and wells were washed again with PBS, 100 µL of oxidant treatment medium (600 µM AAPH and HBSS with 10 mM HEPES) was applied to the cells. Finally, the cellular fluorescence (excitation/emission, 485/535 nm) of the experimental samples (pretreated with samples, AAPH and DCFH-DA), control samples (pretreated with AAPH and DCFH-DA), and blank samples (pretreated with DCFH-DA) was measured every 5 min for 1 h on a microplate reader (EnSpire, Perkin Elmer, Singapore). Quercetin was used as a reference in each experiment, and the EC_50_ values of samples was expressed as mean and converted to CAA values using triplicate separate data. The CAA values were finally expressed as micromoles of quercetin equivalent (QE) per 100 micromoles of RES or PD (μmol QE/100 μmol).

### 3.6. Measurement of Protect Effect against AAPH-Induced Oxidative Stress

The AAPH-induced oxidative stress was determined according to the MTT method reported by Chou et al. [48] with some modifications. Firstly, cells were seeded in a 96-well plate of 2 × 10^4^ cells/well and incubated for 24 h. Then, the cells were treated with different concentrations of AAPH (0.8, 2, 6, 10, 14, 18, 20 mM) and the cell viability was measured by MTT method. After successfully constructing a cell damage model, the cells were treated with the concentration of non-toxic samples for 24 h, and then the suitable concentration of AAPH was added for another 24 h. Finally, the cell viability was determined using MTT method as described above.

### 3.7. ROS Level

ROS generation was evaluated using the method described by Chou et al. [48] with some modifications. Cells were seeded in 12-well plates at a density of 2 × 10^4^ cells/mL in 2 mL complete media for 24 h. Thereafter, the cells were cultured for 24 h with or without RES or PD at different concentrations (0.5, 1, and 2 μg/mL), followed by the addition of AAPH and continued culture at 37 °C for 24 h. Then, cells were incubated with 100 μL of oxidation sensitive dye DCFH-DA (10 μM) at 37 °C for 30 min to monitor the changes in ROS. Finally, cells were washed twice with PBS and immediately measured its fluorescence intensity via a fluorescence microplate reader at 485 nm excitation and 535 nm emission and recorded with a fluorescence microscope.

### 3.8. Measurement of GSH, MDA Levels and Cell Antioxidant Enzyme (LDH, SOD, and CAT) Activity

Oxidative stress in these cells was induced by AAPH. A total of 3 × 10^6^ HepG2 cells were placed in six-well plates. After 24 h of incubation, the medium was discarded and cells were treated with RES or PD at various concentrations (0.5, 1, and 2 μg/mL) for 24 h. After incubation, the cells were treated with AAPH and incubated for 24 h at 37 °C to induce oxidative stress. After the treatment, the supernatants were collected for further determination of LDH, and cell pellets were collected for determination of CAT, SOD, GSH, and MDA immediately. The activities of LDH, SOD, CAT, GSH, and the content of MDA were determined using Assay Kits (Institute of Biological Engineering of Nanjing Jiancheng) according to the protocols. Protein contents were determined by Bicinchoninic Acid Kit (BCA) (BCA; Beyotime, Shanghai, China).

### 3.9. Statistical Analysis

All experiments were carried out at least three times, and the data are presented as mean ± standard error (SE). Significant differences between measurements for the blank, control, and treated samples were analyzed using one-way analysis of variance (ANOVA), followed by Duncan’s post-hoc test (SPSS 17.0).

## 4. Conclusions

This study reported the distribution and contents of RES and PD in Mulberry (*Morus alba* L.). The RES and PD extracted from Mulberry (*Morus alba* L.) confirm that mulberry roots have potential as a source of bioactive compounds and applied to functional food. Furthermore, RES and PD can scavenge various free radicals and enhance the antioxidant defense system of cells. Our discovery establishes insight in understanding the underlying molecular mechanism of RES and PD as antioxidants, and this protective mechanism can lead to the design and development of novel small molecules as potential functional active agents.

## Figures and Tables

**Table 1:** Distribution of RES and PD compounds content extracted from mulberry fruits, mulberry branches, and mulberry roots.

Natural Stilbenes	Mulberry Fruits (μg/g)	Mulberry Branches (μg/g)	Mulberry Roots (μg/g)
RES	0.48 ± 0.0030 ^a^	5.70 ± 0.34 ^c^	32.45 ± 1.72 ^d^
PD	0.0020 ± 0.0028 ^b^	0.33 ± 0.028 ^a^	3.15 ± 1.52 ^c^

Note: Different letters indicate statistically significant differences (*p* < 0.05) among different groups.

## Data Availability

Not applicable.

## Sample Availability

The resveratrol and polydatin are available from the authors.
